# Supplementary material for: Glucosinolates and Isothiocyantes in Processed Rapeseed Determined by HPLC-DAD-qTOF
Source: Plants (Basel). 2021 Nov 22;10(11):2548. doi: 10.3390/plants10112548 (PMC8622084; doi:10.3390/plants10112548)
Supplement: Supplementary file 1 [file plants-10-02548-s001.zip › plants-1456334-supplementary.pdf]

Supplementary

Table S1. The molecular formulas and molar masses of the glucosinolates that were selected according to Tolra et al., 2000 and Metlin Metabolites dataset for identification in the samples. The underlined compounds were identified in this study selected samples.

**Name**	**Formula**	**M (g/moll)**	**Theoretical mass**	**Measured monoisotopic mass**	**Error (ppm)**
3-Methylsulfinylpropyl glucosinolate	$C_{11}H_{20}NO_{10}S_3$	422.5			
4-Methylsulfinylbutyl glucosinolate	$C_{12}H_{22}NO_{10}S_3$	436.5			
6-Methylsulfinylhexyl glucosinolate	$C_{14}H_{26}NO_{10}S_3$	464.6			
7-Methylsulfinylheptyl glucosinolate	$C_{15}H_{28}NO_{10}S_3$	478.6			
4-Methyltiobutyl glucosinolate	$C_6H_{11}NS_2$	421.5	421.0535	421.0544	2.26
4-Hydroxybutyl glucosinolate	$C_{11}H_{21}NO_{10}S_2$	391.1			
2-Propenyl glucosinolate<br>SINIGRIN	$C_{10}H_{17}NO_9S_2$	359.4	359.0345	359.0358	3.79
3-Butenyl glucosinolate<br>GLUCONAPIN	$C_{11}H_{19}NO_9S_2$	373.4	373.0501	373.0512	2.92
2-Hydroxy-3-butenyl glucosinolate<br>PROGOITRIN or<br>(2R)-2-Hydroxybut-3-enylglucosinolate<br>EPIPROGOITRIN	$C_{11}H_{19}NO_{10}S_2$	389.4	389.0450	389.0462	3.11
2-Hydroxy-4-pentenyl glucosinolate					

GLUCONAPOLIFERIN	$C_{12}H_{21}NO_{10}S_2$	403.4	403.0607	403.0621	3.44
Benzyl glucosinolate	$C_{14}H_{18}NO_9S_2$	408.4			
p-Hydroxybenzyl glucosinolate SINALBIN	$C_{14}H_{19}NO_{10}S_2$	425.4	425.0450	425.0458	1.81
2-Phenylethyl glucosinolate GLUCONASTURTIIN	$C_{15}H_{21}NO_9S_2$	423.5	423.0658	423.0670	2.87
3-Indolylmethyl glucosinolate GLUCOBRASSICIN	$C_{16}H_{20}N_2O_9S_2$	448.5			
(N-Sulfoindol-3-yl)methylglucosinolate	$C_{16}H_{20}N_2O_{12}S_3$	528.0			
4-Mercaptobutyl glucosinolate	$C_{11}H_{21}NO_9S_3$	407.0			
2-Hydroxypropyl glucosinolate	$C_{10}H_{19}NO_{10}S_2$	377.0			
Butyl glucosinolate	$C_{11}H_{21}NO_9S_2$	375.1			
6-Heptenyl glucosinolate	$C_{14}H_{25}NO_9S_2$	415.1			
Hexyl glucosinolate	$C_{13}H_{25}NO_9S_2$	403.1			
3-Phenylpropyl glucosinolate	$C_{16}H_{23}NO_9S_2$	437.1			
Methylpentyl glucosinolate	$C_{13}H_{25}NO_9S_2$	403.1	403.0971	403.0994	5.74
2-Methylbutyl glucosinolate	$C_{12}H_{23}NO_9S_2$	389.1			
2-Methylpropyl glucosinolate / Butyl glucosinolate	$C_{11}H_{21}NO_9S_2$	375.1	375.0658	375.0668	2.87

8-Methylthiooctyl glucosinolate	$C_{16}H_{31}NO_9S_3$	477.1			
7-Methylthioheptyl glucosinolate	$C_{15}H_{29}NO_9S_3$	463.1			
4-Methylthiobutyl-desulfoglucosinolate	$C_{12}H_{23}NO_6S_2$	341.1			
3-Methylthiopropyl-desulfoglucosinolate	$C_{11}H_{21}NO_6S_2$	327.1			
Hydroxybenzyldesulphoglucosinolate	$C_{14}H_{19}NO_7S$	345.1			
Indolylmethyl-desulfoglucosinolate	$C_{16}H_{20}N_2O_6S$	368.1			
4-Pentenyl glucosinolate GLUCOBRASSICANAPIN	$C_{12}H_{21}NO_9S_2$	387.1	387.0658	387.0671	3.38
3-Methylpentyl glucosinolate	$C_{13}H_{25}NO_9S_2$	403.1			
(2R)-2-Hydroxy-2-(4-hydroxyphenyl)ethyl glucosinolate	$C_{15}H_{21}NO_{11}S_2$	455.1			
(2R)-2-Hydroxy-2-phenylethyl glucosinolate	$C_{15}H_{21}NO_{10}S_2$	439.1			
2-(Methylthio)ethyl glucosinolate	$C_{10}H_{19}NO_9S_3$	393.0			
6-(Methylthio)hexyl glucosinolate	$C_{14}H_{27}NO_9S_3$	449.1			
6-(Methylsulfonyl)hexyl glucosinolate	$C_{14}H_{27}NO_{11}S_3$	481.1			
3-Methylbutyl glucosinolate	$C_{12}H_{23}NO_9S_2$	389.1			
2-Hydroxypentyl glucosinolate	$C_{12}H_{23}NO_{10}S_2$	405.1			
Pentyl glucosinolate	$C_{12}H_{23}NO_9S_2$	389.1			
4-Phenylbutyl glucosinolate	$C_{17}H_{25}NO_9S_2$	451.1			
5-Hexenyl glucosinolate	$C_{13}H_{23}NO_9S_2$	401.1			

Napoleiferin					
	C_6H_9NOS	143.2			
5-methylsulfinylpentyl glucosinolate					
GLUCOALYSSIN	$C_{13}H_{25}NO_{10}S_3$	451.5	451.0641	451.0658	3.8
1-Methoxy-3-indolylmethyl glucosinolate					
NEOGLUCOBRASSICIN	$C_{17}H_{22}N_2O_{10}S_2$	478.5	478.0716	478.0729	2.8
4-Hydroxy-3-indolylmethylglucosinolate					
4-HYDROXYGLUCOBRASSICIN	$C_{16}H_{20}N_2O_{10}S_2$	464.5			

Table S2. Results of determination of glucosinolates and isothiocyantes in CPR, CPR -2, CPR-3, CPR-4, HPR – 1, HPR – 2 and HPR – 3.

Name of the compound	Formula	M	$[M-H]^-$ / $[M-H]^+$	Confirmation Fragments	Fragment 96 / 97 ratio	Type of Sample	Conc. (mg/kg)
2-Hydroxy-3-butenyl glucosinolate (progoitrin)	$C_{11}H_{19}NO_{10}S_2$	389.0460	388.0389	96.9595<br>259.0183	1 : 2	CPR<br>HPR – 1<br>HPR – 2<br>HPR – 3<br>CPR – 2<br>CPR – 3<br>CPR – 4	1014 ± 56<br>1600 ± 160<br>1720 ± 10<br>2565 ± 3.0<br>< LOD<br>< LOD<br>< LOD
2-Propenyl glucosinolate (sinigrin)	$C_{10}H_{17}NO_9S_2$	359.0287	358.0287	96.9586<br>259.0012	1 : 2	CPR<br>HPR – 1<br>HPR – 2	3.6 ± 0.09<br>< LOD<br>< LOD

						HPR – 3<br>CPR – 2<br>CPR – 3<br>CPR – 4	< LOD<br>< LOD<br>< LOD<br>< LOD
p-Hydroxybenzyl glucosinolate (sinalbin)	$C_{14}H_{19}NO_{10}S_2$	425.0460	424.0387	96.9595<br>259.0106	1 : 4	CPR<br>HPR – 1<br>HPR – 2<br>HPR – 3<br>CPR – 2<br>CPR – 3<br>CPR – 4	21.7 ± 2.2<br>< LOD<br>< LOD<br>< LOD<br>< LOD<br><LOD<br><LOD
2-Hydroxy-4-pentenyl glucosinolate (gluconapoliferin)	$C_{12}H_{21}NO_{10}S_2$	403.0620	402.0549	96.9602<br>259.0130	1 : 3	CPR<br>HPR – 1<br>HPR – 2<br>HPR – 3<br>CPR – 2<br>CPR – 3<br>CPR – 4	114.8* ± 8.0<br>28.3* ± 0.1<br>54* ± 5<br>3.0* ± 0.3<br><LOD<br><LOD<br><LOD
Glucoalyssin	$C_{13}H_{25}NO_{10}S_3$	451.0660	450.0586	96.9602<br>259.0079	1 : 2	CPR<br>HPR – 1<br>HPR – 2<br>HPR – 3<br>CPR – 2<br>CPR – 3	52.1*± 0.04<br>< LOD<br>35.4*± 0.3<br>52* ± 0.3<br><LOD<br><LOD

						CPR – 4	<LOD
3-Butenyl glucosinolate (gluconapin)	$C_{11}H_{19}NO_9S_2$	373.0510	372.0439	96.9601<br>259.0146	1 : 3	CPR<br>HPR – 1<br>HPR – 2<br>HPR – 3<br>CPR – 2<br>CPR – 3<br>CPR – 4	2413 ± 27<br>1479 ± 5.8<br>2024 ± 2.7<br>2209 ± 29<br><LOD<br><LOD<br><LOD
2-methylpropyl glucosinolate / butyl glucosinolate	$C_{11}H_{21}NO_9S_2$	375.0670	374.0598	96.9523<br>259.0247	1 : 3	CPR<br>HPR – 1<br>HPR – 2<br>HPR – 3<br>CPR – 2<br>CPR – 3<br>CPR – 4	16.3* ± 1.1<br>5.3* ± 0.05<br>4.8*± 0.03<br>5.3* ± 0.1<br><LOD<br><LOD<br><LOD
4-Pentenyl glucosinolate (glucobrassicanapin)	$C_{12}H_{21}NO_9S_2$	387.0670	386.0598	96.9601<br>259.0124	1 : 3	CPR<br>HPR – 1<br>HPR – 2<br>HPR – 3<br>CPR – 2<br>CPR – 3<br>CPR – 4	23.7*±1.7<br>16.5* ± 0.08<br>64.0*± 1.0<br>60.9*± 0.2<br><LOD<br><LOD<br><LOD
4-Methyltiobutyl glucosinolate	$C_{12}H_{23}NO_9S_3$	421.0540	420.0472	96.9602<br>259.0137	1 : 3	CPR<br>HPR – 1<br>HPR – 2<br>HPR – 3	< LOQ<br>< LOD<br>< LOD<br><LOD

						CPR – 2<br>CPR – 3<br>CPR – 4	<LOD<br><LOD<br><LOD
2-Phenylethyl glucosinolate (gluconasturtiin)	$C_{15}H_{21}NO_9S_2$	423.0670	422.0597	96.9608<br>259.0075	1 : 3	CPR<br>HPR – 1<br>HPR – 2<br>HPR – 3<br>CPR – 2<br>CPR – 3<br>CPR – 4	22.0 ± 0.8<br>< LOD<br>11.3 ± 0.3<br>28.9 ± 0.4<br><LOD<br><LOD<br><LOD
Methylpentyl glucosinolate	$C_{13}H_{25}NO_9S_2$	403,099	402,0913	96.9627<br>259.0098	1 : 2	CPR<br>HPR – 1<br>HPR – 2<br>HPR – 3<br>CPR – 2<br>CPR – 3<br>CPR – 4	62.7*±4<br>< LOD<br>1.5*±0.05<br>4.1*±0.3<br><LOD<br><LOD<br><LOD
Neoglucobrassicin	$C_{17}H_{22}N_2O_{10}S_2$	478.073	477.0657	96.9605<br>259.0131<br>446.0503	1 : 3	CPR<br>HPR – 1<br>HPR – 2<br>HPR – 3<br>CPR – 2<br>CPR – 3<br>CPR – 4	< LOQ<br>< LOD<br>1.9* ± 0.1<br>10.3* ± 0.2<br><LOD<br><LOD<br><LOD

4-Hydroxybutyl isothiocyanate	C_5H_9NOS	131.0401	132.0470	132.0462		CPR<br>HPR – 1<br>HPR – 2<br>HPR – 3<br>CPR – 2<br>CPR – 3<br>CPR – 4	< LOQ<br>< LOD<br>< LOD<br>< LOD<br>< LOD<br>< LOD<br>< LOD
Goitrin	C_5H_7NOS	129.0240	130.0310	130.0321		CPR<br>HPR – 1<br>HPR – 2<br>HPR – 3<br>CPR – 2<br>CPR – 3<br>CPR – 4	< LOD<br>< LOD<br>< LOD<br>< LOD<br>95 ± 7<br>7 ± 0.5<br>22 ± 1.5
4-Methylthiobutyl isothiocyanate	$C_6H_{11}NS_2$	161.032	179.0630**	103.1010		CPR<br>HPR – 1<br>HPR – 2<br>HPR – 3<br>CPR – 2<br>CPR – 3<br>CPR – 4	< LOD<br>< LOD<br>< LOD<br>< LOD<br>< LOQ<br>< LOD<br>< LOD

2-Phenylethyl isothiocyanate	C_9H_9NS	163.0449	181.0786**	105.0695		CPR<br>HPR – 1<br>HPR – 2<br>HPR – 3<br>CPR – 2<br>CPR – 3<br>CPR – 4	< LOD<br>< LOD<br>< LOD<br>< LOD<br>< LOD<br>< LOQ<br>< LOD
3-Butenyl isothiocyanate	C_5H_7NS	113.0290	131.0600**	114.0328		CPR<br>HPR – 1<br>HPR – 2<br>HPR – 3<br>CPR – 2<br>CPR – 3<br>CPR – 4	< LOD<br>< LOD<br>< LOD<br>< LOD<br>< LOD<br>< LOQ<br>< LOD

*The results were expressed as sinigrin equivalents. **NH_4 adduct. LOD for glucosinolates determination varied from 0.1 mg/kg to 0.37 mg/kg dried weight, while LOQ varied in the range from 1.0 mg/kg to 3.7 mg/kg dried sample. LOD for isothiocyantes determination varied from 0.07 mg/kg sample to 0.42 mg/kg sample, while LOQ varied from 0.7 mg/kg sample to 4.2 mg/kg dried sample.

Table S3. Identification of goitrin determined at different stages of pilot-scale protein extraction from cold-pressed rapeseed cake (CPR).

**Name of the compound**	**Formula**	**M**	**[M-H]$^+$**	**Fragment ion**	**Type of Sample**	**Concentrations (mg/kg d.w.)**
Goitrin	C_5H_7NOS	129.0240	129.0240	130.3190	S3	805 ± 6
					S4	518 ± 36
					S5	528 ± 37
					S6	173 ± 12

					S7	493 ± 35
					S8	52 ± 4

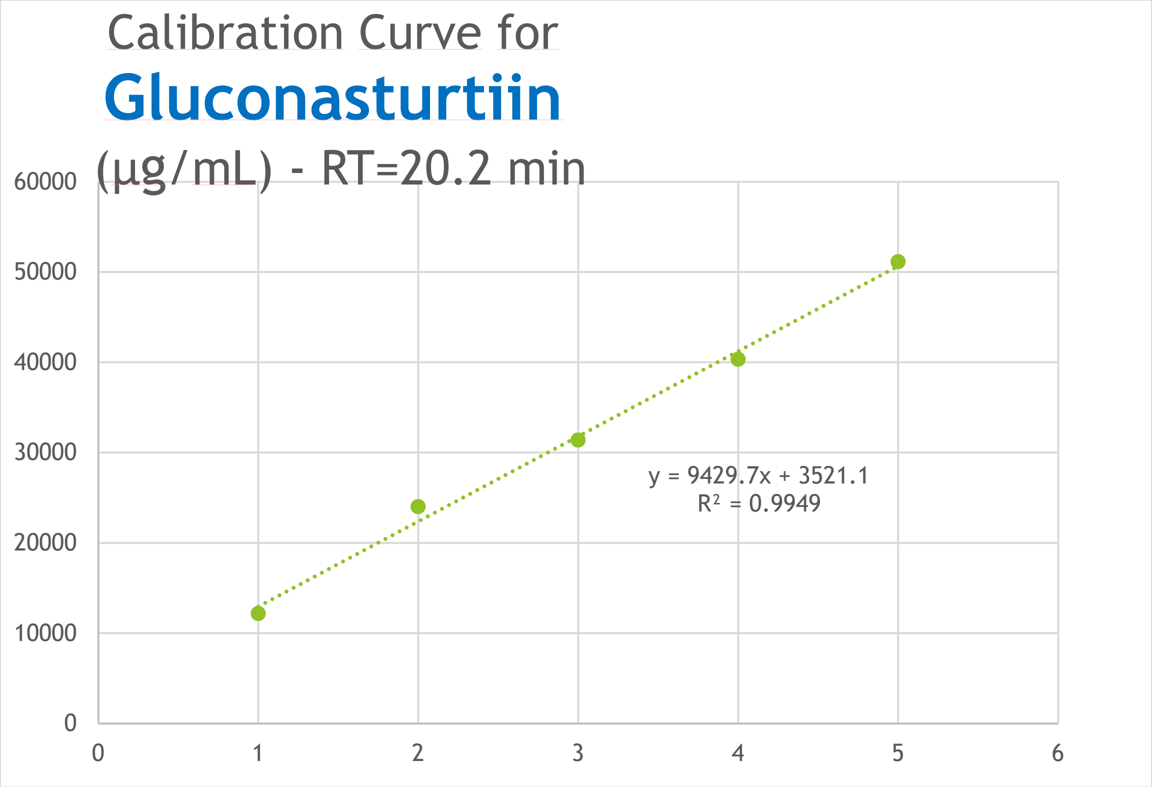

* RT: retention time, y axis – abundances, x axis – concentrations in µg/mL of the spiked standard.

Figure S1. Calibration curve for gluconasturtiin.

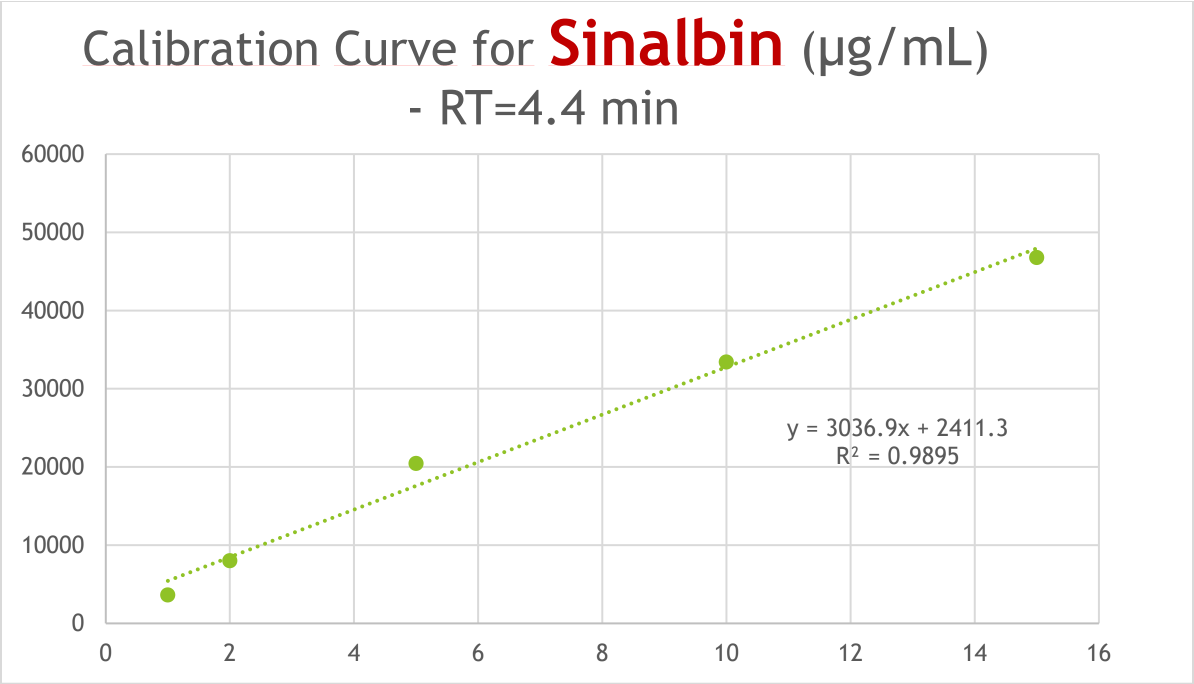

* RT: retention time, y axis – abundances, x axis – concentrations in μg/mL of the spiked standard.

Figure S2. Calibration curve for determination of sinalbin.

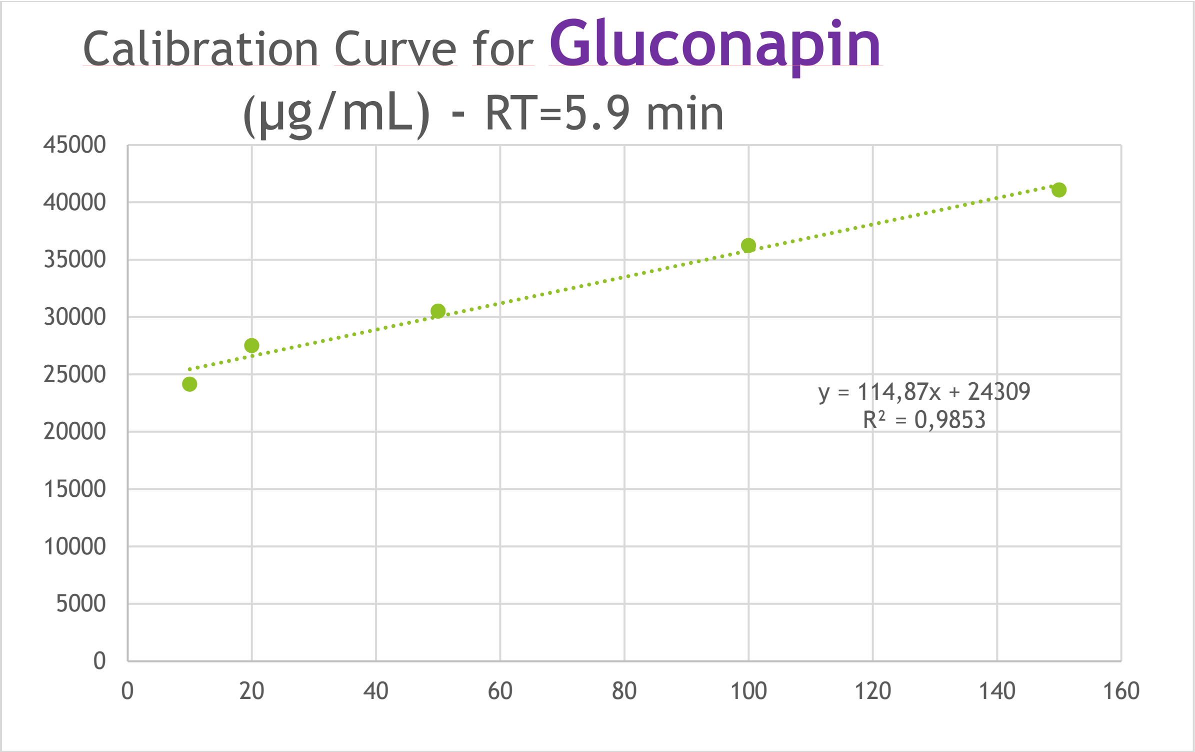

* RT: retention time, y axis – abundances, x axis – concentrations in µg/mL of the spiked standard.

Figure S3. Calibration curve for determination od guconapin.

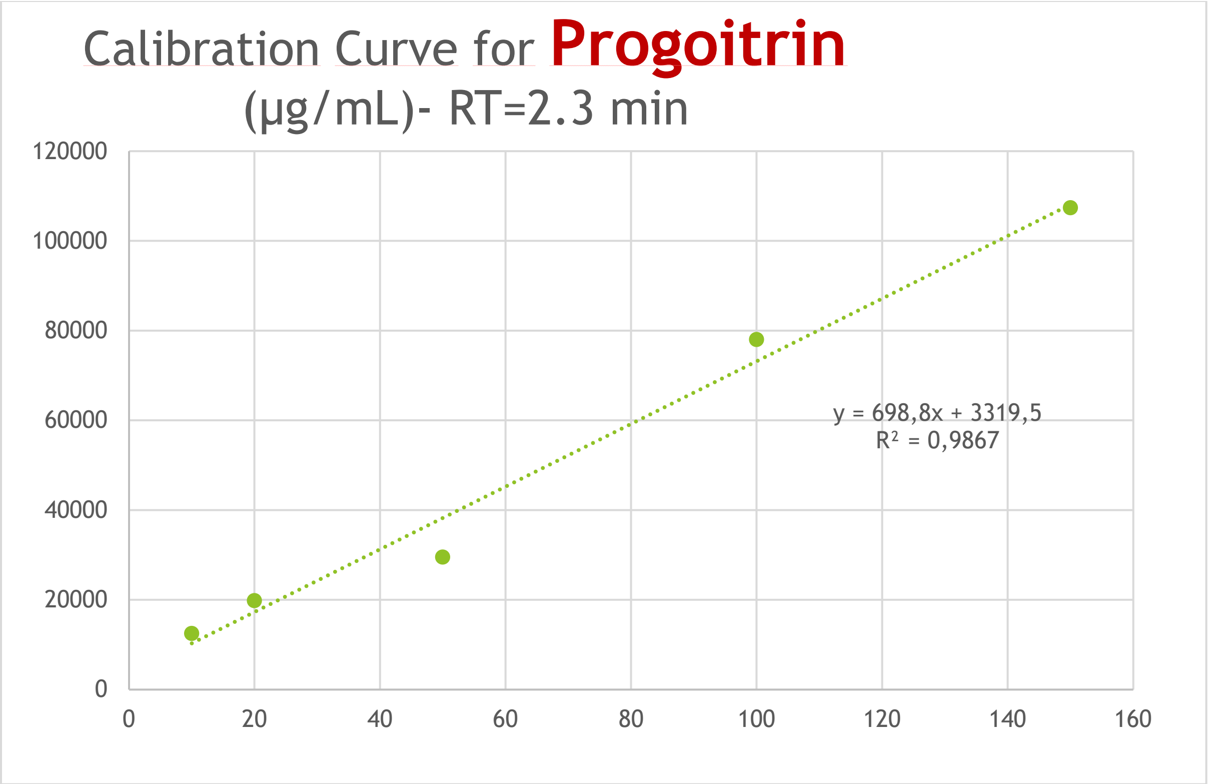

* RT: retention time, y axis – abundances, x axis – concentrations in µg/mL of spiked standard.

Figure S4. Calibration curve for determination of progoitrin.

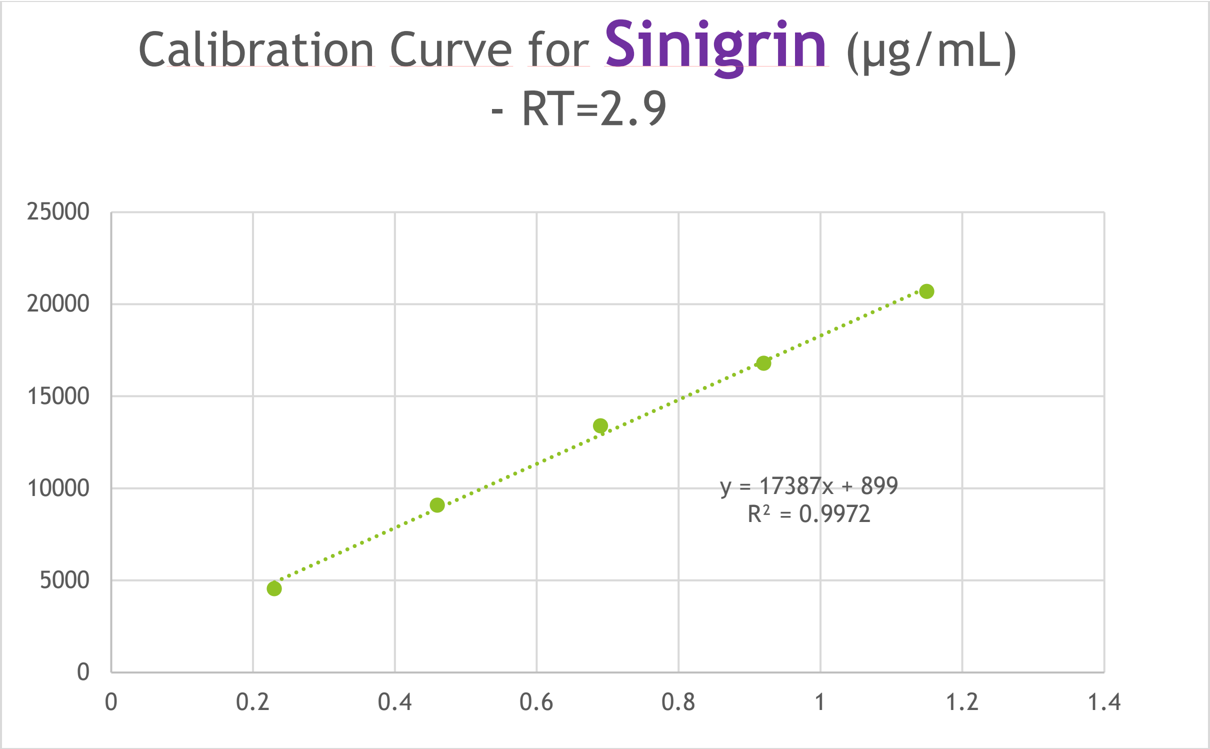

* RT: retention time, y axis – abundances, x axis – concentrations in μg/mL of spiked standard.

Figure S5. Calibration curve for determination of sinigrin and other glucosinolates.
